# Detached self-observation as a developmental catalyst: theoretical insights from dynamical systems thinking, cultural mediation, and contemplative practice

**DOI:** 10.3389/fpsyg.2025.1659774

**Published:** 2025-10-02

**Authors:** Lea Loncar, Charlotte Fiskum

**Affiliations:** Department of Psychology, Norwegian University of Science and Technology, Trondheim, Norway

**Keywords:** detached self-observation, contemplative practices, meditation and mindfulness, dynamical systems theory, cultural psychology, wisdom, neuroplasticity

## Abstract

Detached self-observation, a central dimension of mindfulness meditation, has emerged as a key metacognitive and emotional-regulatory capacity across psychology, education, and neuroscience. This theoretical paper proposes meditation as a developmental catalyst, drawing on contemporary scholarship. Detached observation is reframed here as a culturally mediated competence grounded in both phenomenological embodiment and symbolic internalization. Drawing on recent research in contemplative neuroscience, cultural psychology, dynamical systems theory, and virtue ethics, the paper explores how practices of self-observation foster cognitive-affective flexibility, reflective wisdom, and prosocial behavior. Crucially, the paper also considers potential contraindications, including the risk of depersonalization and cultural misalignment, highlighting the importance of context-sensitive application. Meditation is proposed as a dynamic developmental catalyst, with implications for personal transformation and collective well-being. The paper integrates cultural mediation theory, embodied phenomenology, and transpersonal and dynamical systems perspectives on psychological development. Implications are outlined for educational, therapeutic, and civic contexts across diverse contemplative practices.

## Introduction

The practice of detached self-observation - the capacity to observe one’s thoughts, emotions, and internal states without immediate identification or reactivity - has emerged as a central construct in contemporary psychology, particularly within mindfulness research, developmental theory, and contemplative science (Carlson, 2013; Dorjee, 2016; Shapiro et al., 2006). In this paper, meditation is foregrounded as the primary context in which detached self-observation is cultivated. Although often associated with Buddhist meditation, yoga, or therapeutic practices such as mindfulness-based cognitive therapy, detached self-observation has deep historical and philosophical roots that intersect with both Western and non-Western frameworks of personal transformation (Feuerstein, 1998; Walsh, 1999). As outlined in Appendix A, comparable modalities of detached awareness have been recognized across cultural traditions - from Buddhist sati and Vedāntic sakshi caitanya, to Sufi muraqaba, Indigenous vision quests, and African Ubuntu practices - indicating that detached self-observation is a recurring and transformative human experience. This paper presents a comprehensive lens that incorporates insights from recent cultural psychology, contemplative neuroscience, virtue ethics, and dynamical systems theory (DST) in order to understand meditation’s role in cognitive, emotional, and social flourishing.

Historically, contemplative traditions have viewed observation of the self as the basis for liberation, wisdom, and integration (Taimni, 1993; Tart, 1990). In early Buddhist texts, mindfulness (sati) is described as a tool for insight and ethical discernment. Similarly, in the Yoga Sutras of Patanjali, svadhyaya (self-study) is a foundational discipline for internal transformation. Furthermore, Patanjali considers pratyāhāra (withdrawal of the senses) a key practice for deepening detached awareness and developing insight into the nature of the self. These ancient perspectives resonate with humanistic and existential psychology in the West, particularly in the writings of Maslow (1971), May (1981), and Deikman (1982), who framed peak experience, existential presence, and the “observing self” as markers of psychological maturity. With the increasing scientific interest in mindfulness and self-regulation, these practices are being reframed through empirical and neurocognitive lenses (Grossmann et al., 2020; Poublan-Couzardot, 2022).

In developmental science, self-observation has long been recognized as a key capacity enabling reflective thought, emotional regulation, and social adaptation (Tesser et al., 2000; Vygotsky, 1978). Vygotsky’s model of internalization laid the foundation for understanding how culturally mediated tools shape higher mental functions. More recently, cultural-historical theorists such as Valsiner (2000, 2007, 2014) have emphasized the dynamic regulation of internal dialogue, arguing that the development of the self involves both semiotic mediation and active meaning-making. Within this framework, meditation can be viewed not as a withdrawal from the world but as an advanced tool of self-regulatory development and meaning creation, where detached self-observation offers the reflective space in which new symbolic interpretations of experience can develop (Ryan and Deci, 2001).

This paper is a theoretical contribution that positions detached self-observation as a developmental mechanism embedded in symbolic, phenomenological, and social systems. Unlike approaches that treat mindfulness as a static trait or discrete intervention, this model proposes that contemplative practices foster a learnable and culturally mediated form of meta-awareness - one that supports the emergence of wisdom, virtue, and social intelligence over time. Throughout the article, detached self-observation is presented as the unifying thread that connects regulation, meaning-making, and cultural expressions of contemplative awareness. It ultimately emerges as a developmental catalyst and foundation for wisdom. To explore this proposal systematically, the article is structured around four guiding questions, as follows:

Four main questions structure the paper:

1. What is detached self-observation, and how does it develop across different contexts?
2. How does this capacity support personal transformation, including emotional regulation and identity growth?
3. What role does detached observation play in fostering interpersonal understanding and ethical engagement?
4. How can phenomenology, dynamical systems theory, and cultural psychology deepen our understanding of contemplative development?

Each section integrates both classical sources and recent empirical literature to offer a nuanced framework for understanding the developmental power of meditative self-awareness.

## Theoretical framework: detached self-observation as a developmental tool

Detached self-observation can be defined as the sustained capacity to monitor one’s internal experiences - thoughts, emotions, impulses - without immediate identification, judgment, or reaction (Bishop et al., 2004; Lutz et al., 2015). It allows the individual to take a reflective stance, stepping back from experience in order to regulate responses and integrate meaning. While popular mindfulness literature often reduces this to a present-moment orientation, deeper theoretical framing situates detached observation as a complex developmental achievement, emerging from the interplay between culture, embodiment, symbolic regulation, and neurocognitive maturation. Detached self-observation can be distinguished from several related concepts found in contemplative science and psychology. Decentering involves recognizing thoughts as temporary mental events rather than facts (Teasdale et al., 2002). Meta-awareness focuses on monitoring one’s own default cognitive processes (Schooler et al., 2011), while re-perceiving emphasizes a shift in perspective that alters one’s relationship to internal experience (Shapiro et al., 2006). The “observing self,” described in transpersonal psychology (Deikman, 1982; Assagioli, 1974), points to a witnessing aspect of consciousness that transcends ego identification. Detached self-observation, as discussed in this article, combines these dimensions within a developmental and cultural framework. It functions as both a moment-to-moment practice and a learnable skill that promotes regulation, meaning-making, and ultimately, wisdom, within a cultural context.

From a cultural-historical perspective, detached self-observation is best understood as a symbolically mediated function. From this perspective, reflective awareness does not occur in isolation, but is shaped by culturally embedded signs, practices, and symbols that guide how inner processes are observed and interpreted (Vygotsky, 1978; Valsiner, 2007). This becomes apparent when exploring cultural traditions that, while diverse, have each articulated forms of detached awareness in strikingly similar ways. In Vedānta, the concept of sakshi caitanya (“witnessing consciousness”) describes the self as an impartial observer of mental states (Feuerstein, 1998), while Yoga highlights pratyāhāra (withdrawal of the senses) as a path to inner detachment (Taimni, 1993). In Buddhism, mindfulness (sati) and insight into non-self (anattā) foster the same attitude of observation without identification (Anālayo, 2015), whereas Zen practice of zazen deepens this into observing the fabric of thoughts itself, described by Dōgen as “thinking non-thinking” (hishiryo) (Suzuki, 2019; Kasulis, 1978). In Christian mysticism, the idea of nepsis (watchfulness) points to a similar vigilance over internal processes (Lossky, 1957; Casiday, 2006), while in Islamic Sufism, muraqaba refers to meditative witnessing of the heart (Knysh, 2010). Indigenous traditions also express similar modalities: the Lakota vision quest as a discipline of observing mind and nature (Deloria, 2006), Aboriginal practices of dadirri (deep listening) as a receptive stance toward experience (Yunkaporta and Kirby (2011), and the African philosophy of Ubuntu, which combines reflective awareness of self with recognition of relational interdependence (Ramose, 1999). Appendix A provides some examples that vary in metaphysical assumptions but share the common view of detached self-observation as a recurring human experience across cultures. As Vygotsky (1978) 1981 proposed, higher mental functions develop through cultural tools - most notably language - that allow individuals to internalize external regulation. Initially scaffolded by social interaction, these tools become internalized as self-regulatory mechanisms. Meditation, when seen through this lens, is a structured internal dialogue - one that replaces reactive thought with intentional observation (Valsiner and Valsiner, 1997; Valsiner, 2000).

According to Valsiner (2007), the self is not a static entity but a dynamic semiotic system shaped by ongoing interactions with signs and symbols in its environment. Meditation can be seen as a semiotic intervention, where practitioners use meaningful clues or “signs”; (e.g., the breath, mantras, bodily sensations) to anchor attention and modulate inner dialogue. Detached self-observation thus arises from a recursive loop between attentional discipline and symbolic transformation - a dynamic Valsiner (2014) describes as “autoregulation of the self.”

Furthermore, cultural variability shapes how detached observation is developed. Some societies scaffold self-observation through collective rituals (e.g., silent retreats, spiritual mentoring), while others emphasize cognitive reframing or moral discipline (Cavanna et al., 2023; Eeles and Walker, 2022; Lutz et al., 2015). Therefore, the developmental arc of self-observation is not universal but deeply context-dependent and culturally embedded (Holland and Skinner, 1997; Tudge et al., 1997).

## Embodiment and phenomenological awareness

From a phenomenological standpoint, detached self-observation is an embodied mode of experience rather than a purely cognitive function. Merleau-Ponty (1962) argued that perception is always mediated through the lived body (le corps propre), which serves as the horizon for any form of consciousness. Meditative practice highlights this insight by using bodily anchors–breath, posture, sensations–not only as objects of attention but as gateways to a non-reactive presence. From a Buddhist perspective, such embodied observation reveals fundamental insights into impermanence (anicca), the absence of an enduring self (anattā), and the interdependent emptiness of phenomena (śūnyatā). In this way, detached self-observation does not reinforce the illusion of a fixed observer but instead reveals the constructed, fluid, and transient nature of experience (Anālayo, 2015; Suzuki, 2019). This perspective parallels Husserl’s (1982) concept of epoché, the suspension of the natural attitude to observe phenomena as they present themselves, free from habitual assumptions. It also aligns with Merleau-Ponty’s (1962) phenomenology, which views embodiment as the fundamental basis for perception. Detached self-observation can be seen as a lived enactment of epoché: a bracketting of automatic identification that creates space for new meaning and a renewed sense of relational awareness. Meditation cultivates this embodied awareness, often by focusing attention on breath or sensation, thus grounding reflection in the immediacy of the body.

Building on this, Varela et al. (1991) introduced the concept of enactive cognition, where mind and world are co-constructed through embodied action. Detached self-observation, in this view, is not a passive mental state but a dynamic stance of openness that alters the structure of experience. Rather than stepping back from the world, the observer becomes more fully present within it. Giorgi (1983) and Deikman (1982) emphasized that this shift is qualitative: it allows a person to perceive thought not as identity but as object. The “observing self” is not separate from the body or the world, but embodied and embedded within an unfolding field of awareness.

## Dynamical systems, altered states, and psychological regulation

Dynamical systems theory provides a powerful framework for understanding meditation and detached self-observation. DST highlights how complex systems self-organize into stable patterns, or attractors, through recursive feedback loops (Kelso, 1995; Thelen and Smith, 1994; Valsiner, 2014). In psychological terms, attractors describe tendencies wherein thought, attention, and emotion stabilize into enduring configurations. Meditation can be seen as a process that reshapes these patterns. Anchors such as breath, mantra, or bodily sensations serve as semiotic cues that guide attentional trajectories and reorganize internal dialogue, thought, and emotion. Detached self-observation plays a key role here serving as a developmental catalyst by creating reflective distance, thereby enabling practitioners to notice emerging attractors without becoming rigidly identified with them.

Valsiner (2007) suggests that human development occurs through perturbation–reorganization loops, where the system adjusts to novelty by creating more complex regulatory structures. In dynamical systems terms, mental and neural processes tend to stabilize around certain attractor states - patterns of thought, feeling, meaning, or connectivity that the system returns to over time (Globus and Arpaia, 1994; Lewis, 2005). Development involves not just the consolidation of such attractors, but also their perturbation (disturbance) and reorganization, which allows the system to transition toward new, more adaptive configurations in response to experience and context. Meditation provides such perturbations by interrupting habitual reaction patterns and supporting adaptive reorganization around new symbolic attractors. Detached self-observation further facilitates this reorganization, enabling the practitioner to generate new meanings rather than defaulting to established patterns.

Detached self-observation is therefore best understood as a recursive process: it enables insight, which supports reflection, which refines future awareness. Through repeated practice, this loop acts as a developmental catalyst fostering emotional balance, cognitive flexibility, and moral clarity.

From a neurodevelopmental perspective, such recursive loops correspond with the self-organizing nature of brain development, in which emotional and attentional states recurrently shape and stabilize patterns of neural activation. Lewis (2005) proposes that corticolimbic configurations that recur frequently in real time become developmentally entrenched through recursive synaptic modification. In other words, the emotional and attentional states we return to most often literally sculpt the brain over time, reinforcing certain neural pathways while making others less accessible. This mechanism provides a neurobiological substrate for how practices like meditation, through repeated cultivation of non-reactivity and meta-awareness, may modulate the very structures that give rise to attention, appraisal, and emotion. Emotional states, in this model, actively shape attractor landscapes, which also explains how meaning is created and stabilized within the system. In line with this, “meaning” is not a fixed code but the organism’s ongoing process of sense-making, in which patterns of perception, categorization, and action are organized into attractor landscapes. These landscapes further bias how later states are selected and stabilized. Through learning and experience, both brain and psyche are gradually sculpted so that certain interpretive tendencies, such as compassionate or threat-focused appraisals, become easier to enter and harder to leave. Within this frame, detached self-observation, or “witnessing” (Sākṣī Caitan̄ya), functions as a recurrent, precision-modulating perturbation or state. It helps to smooth out maladaptive patterns and creates a form of systemic stability around meta-awareness, allowing reinterpretation and reflection rather than reflex or stagnation.

Empirical studies further support this view. Mindfulness meditation has been shown to induce structural and functional changes in brain regions implicated in self-regulation, including increased cortical thickness in the prefrontal cortex, insula, and limbic areas (Lazar et al., 2005, Hölzel et al., 2011; Sevinc et al., 2021). These changes mirror the dynamical principle that stable traits emerge through repeated state activations. Especially during developmental transitions, such structured perturbations can scaffold reorganization toward more integrative configurations.

Thus, meditation may not only facilitate momentary coherence but also serve as a long-term developmental regulator, gradually refining patterns of attention, emotion, and self-reflective reasoning. Detached self-observation is the mechanism that allows these shifts to consolidate, ensuring that short-term states are integrated into enduring developmental traits. Recent neuroimaging strengthens this view. Yue et al. (2023) demonstrated that mindfulness training increased reconfiguration efficiency across the executive control, salience and the default mode networks. These networks are respectively associated with goal-directed attention, interoceptive and emotional awareness, and self-referential processing. Crucially, participants’ brain activity after mindfulness training shifted closer to configurations typical of mindful awareness. This suggests that regular meditation may reduce the neurocognitive “effort” required to enter states of detached self-observation. From a DST perspective, these changes represent a recalibration of the attractor landscape, where mindfulness disrupts habitual patterns and guides the brain toward more adaptive, self-regulatory configurations. These findings suggest that meditation not only stabilizes transient states of awareness but may also reshape long-term patterns of cognitive and emotional processing. Through repeated practice, detached self-observation engages attentional, regulatory, and interoceptive networks in ways that support more adaptive trajectories of development.

Tart (1990) and Walsh (1999) describe how contemplative traditions systematically induce non-ordinary states of consciousness that decentre the ego and expand reflective capacity. Such altered states can be understood in DST terms as temporary attractor states: structured yet fleeting reorganizations of awareness. Detached self-observation plays a key role in transforming these altered states into enduring traits of regulation and meaning-making, ensuring that momentary experiences of calm, insight, or expansion are integrated into the developmental repertoire.

Contemporary neuroimaging supports these interpretations. Studies show that meditative states are associated with downregulation of the default mode network (DMN), associated with self-referential rumination, and upregulation of executive and salience networks, associated with attentional control (Poublan-Couzardot, 2022; Lutz et al., 2015). This neurodynamic reorganization is often subjectively experienced as spaciousness, presence, or decentring - qualities central to detached observation. DMN is thought to sustain habitual self-narratives and self-referential processes. Meditation appears to weaken the dominance of these loops, making room for more flexible, less ego-involved modes of awareness (Lutz et al., 2015). This shift may provide a neurophenomenological basis for the developmental movement from identification to witnessing, highlighting detached self-observation as the mediator of such transformation.

The described integration resonates with Wilber’s (1997) perspective on meditation in transpersonal psychology, as it encompasses both a process of state training and structural development. According to Wilber’s integral model, meditation initially induces non-ordinary states of consciousness, but with repeated practice, these states solidify into stable developmental structures. Detached self-observation can thus be seen as the cross-level integrator: the faculty that allows altered states to influence structural transformation by linking phenomenological experience with systemic attractor dynamics.

## Wisdom as a developmental and systemic attractor

The development of wisdom and virtue represents one of the most enduring goals of contemplative traditions, philosophical inquiry, and psychological development. Detached self-observation, as cultivated through meditative practices, plays a central role in this process by enabling individuals to distance themselves from reactive thought patterns and instead reflect on deeper values, ethical frameworks, and long-term consequences.

In recent psychological literature, wisdom is no longer viewed as a rare trait, but rather as a dynamic developmental capacity that involves the integration of cognitive, emotional, and moral aspects (Verhaeghen, 2020). Sternberg (1990) defines wisdom as the use of intelligence and experience to achieve a common good over the long term. Grossmann et al. (2020) further conceptualize wisdom as comprising five core features: intellectual humility, perspectival metacognition, emotional regulation, prosocial intentions, and the ability to manage uncertainty.

Detached self-observation supports these components by creating a reflective distance, which reduces cognitive bias, softens rigid self-concepts, and facilitates ethical reasoning (Shapiro et al., 2006; Trapnell and Campbell, 1999). Maslow (1971) similarly suggested that reflective awareness can give rise to a “peak-experiencing self,” one that combines critical reflection with openness to transcendent experience.

In contemplative contexts, wisdom is not abstract knowledge but lived discernment, shaped by years of meditative cultivation. For example, in yoga and Buddhist psychology, wisdom (prajñā) is inseparable from ethical clarity (śīla) and attentional mastery (samādhi) (Feuerstein, 1998; Taimni, 1993). From a dynamical systems viewpoint, wisdom emerges as an attractor state: a stable, self-organizing pattern of regulation and meaning-making (Wagoner et al., 2021). Through recursive loops of detached self-observation, practitioners reconfigure attractor landscapes, shifting from reactive conditioning toward reflective awareness and ethical discernment. This process explains how meaning and wisdom can be generated: attractors are not only stabilizing tendencies, but developmental “meaning fields” reorganized through observation and reflection.

Whereas wisdom engages the reflective dimension of consciousness, virtue pertains to the alignment of this reflection with action. Detached self-observation fosters the cultivation of virtues such as patience, humility, compassion, and courage, not by imposing moral codes, but by expanding the field of awareness from which ethical behavior can arise (Kristeller and Jordan, 2018; Martin, 2019). Over time, this practice facilitates the embodiment of virtue as a lived disposition. Compassion-based practices such as metta and karuṇā meditation illustrate this process, showing how detached awareness fosters prosocial attitudes and strengthens relational wisdom (Fredrickson et al., 2008; Singer and Klimecki, 2014).

Developmental models further support this trajectory: Wade’s (1996) holonomic model describes contemplative growth as a progression through increasingly integrated stages of awareness, where moral reasoning is guided less by social convention and more by intuitive, transpersonal insight. Similarly, Murphy (1992) argued that contemplative development fosters not only cognitive expansion but also somatic transformation - a re-tuning of the whole organism toward coherence and compassion.

The recursive practice of detached self-observation gradually shifts the system’s center of gravity from reactive conditioning to reflective awareness. This transformation is non-linear and often marked by crisis, confusion, and restructuring, which are common in both meditative development and ego-stage transitions (Churchill, 2018; Giorgi, 1983) as well as state-shifts in complex systems (Bertuglia and Vaio, 2005). Zittoun and Gillespie (2015) argue that imagination plays a crucial role in this process. Supportive research further indicates that contemplative practices such as loving-kindness meditation can broaden individuals’ capacity to consider alternative identities, moral perspectives, and relational outcomes (Fredrickson et al., 2008; Singer and Klimecki, 2014; Immordino-Yang et al., 2012). Meditation, therefore, expands the horizon of the self, enabling perspective-taking and moral imagination and opening a space where new psychological configurations - more coherent, integrated, and virtuous - can emerge.

This emergent space bears resemblance to Winnicott’s (1991/1971) notion of the potential space, an intermediate area of experiencing where inner and outer realities can be creatively negotiated. Just as the child’s play enables integration of subjective needs with external and contextual demands, meditation cultivates a contemplative potential space where reactive patterns may soften and new forms of being can be experimented with. This space is not merely introspective; it can also be highly relational and symbolic, allowing for the imaginative reworking of self–other dynamics and moral understanding. Compassion-based practices such as metta and karuṇā meditation illustrate this by cultivating personal growth and reshaping one’s stance toward others (Fredrickson et al., 2008). In this sense, detached self-observation functions as the generative matrix of symbolic transformation, where existential tensions can be held and examined rather than prematurely resolved, and where wisdom crystallizes as a new attractor influencing one’s orientation toward life, others, and self.

## Detached self-observation beyond the self: relational, ethical, and social flourishing

While detached self-observation is often framed as an inward-facing skill, its relational and social consequences are profound. The ability to witness one’s thoughts, impulses, and emotions without automatic identification enhances interpersonal sensitivity, reduces reactivity, and supports ethical behavior in social contexts. This section explores how detached observation shapes relational maturity, leadership, and civic engagement, linking intrapersonal insight with broader flourishing.

Mindfulness research has consistently shown that non-reactive attention cultivates emotional intelligence, empathic concern, and interpersonal effectiveness (Rupprecht et al., 2019; Shapiro et al., 2006). Detached self-observation allows individuals to interrupt defensive routines, such as projection, withdrawal, or blame, thereby opening space for reflective communication. Carlson (2013) noted that mindfulness increases alignment between perceived and actual self-knowledge, a crucial prerequisite for authentic relationships. Similarly, Sedikides and Skowronski (1995) proposed that self-reflection is a prerequisite for self-knowledge.

This capacity is especially relevant in emotionally charged interactions, where automaticity often drives conflict. Practitioners who develop a habit of detached observation are more likely to respond with clarity and curiosity rather than defensiveness or aggression. This parallels Heidegger’s (1962) notion of presence as an «openness-to-being-with-others», as well as Merleau-Ponty’s (1962) view of embodied intersubjectivity.

Relational awareness is not simply about managing one’s responses - it also entails an ethical stance of humility and respect, grounding self-regulation in a relational ethic. As Kristeller and Jordan (2018) argued, contemplative practice facilitates other-centered perspective-taking, a shift from egoic defensiveness to compassionate engagement.

These interpersonal skills extend into leadership and civic responsibility. In leadership contexts, detached self-observation fosters self-regulation, perspective-taking, and principled decision-making - qualities increasingly valued in both corporate and educational sectors (Frizzell, 2021). Mindful leaders demonstrate “low-reactivity/high-clarity” presence, enabling them to guide others without imposing control or retreating into avoidance.

According to Sternberg (1990) and Grossmann et al. (2020), wise leadership involves balancing competing interests through metacognitive awareness and moral integrity. Meditation contributes to this by supporting a dialectical stance: the ability to hold multiple viewpoints, acknowledge complexity, and act without rigid attachment to egoic goals.

Trapnell and Campbell (1999) highlighted how constructive self-awareness underpins authentic communication. Detached observers are more capable of admitting error, adjusting course, and relating to others without defensiveness - key capacities for dialogic engagement. These relational skills are increasingly critical in times of polarization and ecological crisis (Smith, 2017; Walsh, 1999).

At the societal level, detached observation supports what might be termed “contemplative citizenship” - an ethic of awareness, humility, and moral responsiveness within civic life (Talisse, 2024). Mindfulness-based education and community practices have shown promise in reducing aggression, increasing pro-social behavior, and strengthening intergroup empathy (Churchill, 2018; Sulianta, 2024). These outcomes suggest that contemplative capacities, once seen as private virtues, may have broad public value.

Detached self-observation also enables resistance to collective conditioning. By stepping back from inherited narratives, individuals gain the freedom to choose alternative roles, values, and identities (Tesser et al., 2000; Vygotsky, 1981), as in Winnicott’s potential spaces. Belenky et al. (1997) argued that this form of self-authorship is particularly important for marginalized voices, whose socialization often suppresses epistemic agency.

Moreover, this awareness is shaped by cultural context. As Moye et al. (2025) demonstrate, the internalization of contemplative reflection varies significantly across collectivist and individualist cultures. In some settings, self-observation is oriented toward harmony and humility; in others, it fosters autonomy and authenticity. This cultural embeddedness underscores the systemic implications of detached self-observation as both a personal and collective practice. Taken together, the relational, ethical, and civic dimensions suggest that detached self-observation could act as a developmental catalyst, connecting individual reflection with the cultivation of wisdom, virtue, and social flourishing.

## Phenomenological and methodological foundations of detached self-observation

Any theoretical account of meditation and human development must address not only what develops, but how we know what development means. In this regard, phenomenology offers a crucial foundation. Unlike positivist paradigms, phenomenology seeks to understand the structure of lived experience, asking how meaning arises through the dynamic relation between the self, the body, and the world (Giorgi, 1983; Merleau-Ponty, 1962). Detached self-observation, in this view, is not merely a technique but a modality of being - a disciplined way of inhabiting and reflecting on one’s experiential world.

Giorgi (1983) argued that phenomenological psychology is particularly well suited to study meditative states because it respects both the subjective and the intentional dimensions of consciousness. Detached observation reveals the contents of awareness as they present themselves, without reducing them to underlying variables. This kind of inquiry aligns with Deikman’s (1982) call for a psychology of the “observing self,” one that takes interiority seriously without psychologizing it into pathology.

The act of observing one’s thoughts - without fusing with them - is a primary datum in both meditation and phenomenology. Giorgi saw this as a methodologically rigorous stance, akin to the phenomenological epoché, or suspension of belief. In this light, meditation could be seen as a form of applied epoché: a repetitive, embodied discipline of bracketed observation that alters the structure of the self-world relation.

Extending this work, Varela et al. (1991) proposed neurophenomenology as a framework that integrates first-person experience with third-person neuroscience. This interdisciplinary model suggests that lived experience can inform and constrain models of cognition, provided that it is accessed through trained, systematic introspection - precisely the kind cultivated in long-term meditation. Detached self-observation, then, is not just a topic for psychological theory but a method of psychological inquiry. It enables practitioners to become epistemological subjects - observers of their own cognitive and affective processes - contributing to what Varela called a “first-person science of consciousness.”

From a developmental lens, contemplative practice can also be situated in postformal and holonomic theories of consciousness. Wade (1996) proposed that contemplative practice opens access to postformal stages - levels of awareness marked by paradox tolerance, perspectival fluidity, and ethical coherence. The model describes a progression through increasingly integrated stages of awareness, where moral reasoning shifts from social convention to transpersonal insight. Churchill (2018) and Maslow (1968) likewise emphasized that mature development often involves trans-egoic states, often catalyzed by reflective or peak experiences. Murphy (1992) further argued that contemplative growth is not only cognitive but also somatic, a re-tuning of the organism toward coherence and compassion. Seen through the lens of such proposed models, meditation is not merely stress reduction or attentional training, but a scaffold for advanced human development and self-understanding.

At the same time, it is important to acknowledge potential limitations and risks. Detached self-observation, when misapplied, may risk reinforcing dissociative tendencies or depersonalization, particularly in populations with unresolved trauma or psychiatric vulnerability (Ciaunica et al., 2023; Sass et al., 2013; Jung, 1939). Notably, Carl Jung warned as early as the 1930s that Eastern contemplative techniques may not be appropriate and can even be dangerous for Western contexts without cultural translation, a point echoed by Britton (2019) who cautioned against homogenizing diverse contemplative practices under the umbrella of “mindfulness.” These insights underscore the need for pluralism in contemplative science, where practices are embedded in cultural, clinical and developmental contexts.

## Final remarks and implications

This paper has re-examined detached self-observation not as a narrow stress-reduction technique, but as a developmental and cultural mechanism embedded in contextual, symbolic, and embodied processes. Integrating insights from contemplative science, cultural psychology, virtue ethics, phenomenology, and DST, the paper reframes meditation as a multi-level developmental catalyst - a symbolic practice that cultivates reflective self-regulation, ethical maturity, and interpersonal depth.

Detached self-observation supports psychological flexibility, emotional integration, and wisdom, not by suppressing thought or emotion, but by altering the stance from which they are experienced. It enables a functional decentring - what Shapiro et al. (2006) call “reperceiving” - which fosters self-inquiry, moral clarity, and ethical alignment. As shown across traditions, from Sākṣī Caitan̄ya in Vedānta to dadirri in Aboriginal practice, such detached awareness represents a recurring human capacity, culturally expressed in diverse ways. These processes are not esoteric; they are learnable, culturally embedded, and empirically observable through both first-person and neurocognitive methods (Varela et al., 1991; Poublan-Couzardot, 2022).

Importantly, this paper positions meditation as a culturally scaffolded, context-sensitive practice. Detached self-observation develops differently across settings, influenced by language, social norms, and available tools of reflection (Valsiner, 2007; Moye et al., 2025). Its developmental trajectory is recursive and non-linear, shaped by feedback loops between states and traits, where altered states may stabilize into attractor patterns of meaning and wisdom.

The implications of this framework are vast and applicable in various domains. In the field of education, contemplative pedagogy can enhance meta-cognition, emotional literacy, and intercultural understanding (Churchill, 2018). Within therapeutic contexts, mindfulness practices are not only beneficial for symptom relief but also for identity reconstruction, trauma integration, and moral repair (Dorjee, 2016). In civic life, detached awareness encourages critical thinking, non-reactive dialogue, and democratic deliberation (Smith, 2017; Walsh, 1999). Moreover, in the realm of adult development, meditation can facilitate postformal reasoning, transpersonal growth, and the cultivation of embodied wisdom (Wade, 1996; Maslow, 1968). As crises of meaning, fragmentation, and reactivity grow in scale, the cultivation of reflective awareness becomes not only a personal project but a collective necessity. Detached self-observation - far from being a withdrawal from life - is a return to it, fully present, ethically grounded, and developmentally oriented.

## Data Availability

The original contributions presented in this study are included in this article/supplementary material, further inquiries can be directed to the corresponding author.

## Appendix

**Appendix A:** Cross-cultural and theoretical parallels to detached self-observation.

Tradition/domain	Core practice/concept	Description	Representative figures/sources
Indian – Yoga (Pātañjala)	*Pratyāhāra*, *Kaivalya*	Withdrawal of senses; detachment from reactive mental patterns to realize pure consciousness	Patañjali (*Yoga Sūtras* II.54–55); Taimni (1993)
Indian – Vedānta (Advaita)	*Sākṣī Caitan̄ya*, *Neti-Neti*	Non-dual witnessing awareness; realization of Self as Brahman beyond ego	Śaṅkara (8th c.); Feuerstein (1998)
Buddhism and Zen	*Sati*, *Anattā*, *Hishiryo*	Mindful, non-reactive awareness; detachment from fixed identity	Anālayo (2015); Thera (2005); Suzuki (2019); Kasulis (1978)
Christian Mysticism	*Nepsis*, *Hesychasm*	Watchfulness over thoughts; cultivating inner stillness and humility	Casiday (2006) Evagrius Ponticus (4th c.); Lossky (1957)
Islamic Sufism	*Muraqaba*, *Tafakkur*	Meditative vigilance; observing the self and transcending the ego	Al-Ghazali (11th c.); Knysh (2010)
Toltec (Mesoamerican)	*Second Attention*	Shifting perception beyond social programming to reach deeper awareness	Nelson and Ruiz (1997); Castaneda (1971)*
Southern Africa (Ubuntu)	*Reflective Ethics*	Selfhood through moral reflection in relational and communal context	Ramose (1999)
Aboriginal Australia	*Dreaming*, *Deep Listening (dadirri)*	Interconnected awareness via land, story, and ancestors	Yunkaporta and Kirby (2011)
North American Indigenous	*Vision Quest* (*Hanbleceya*)	Solitary fasting for insight and symbolic rebirth; ego detachment	Deloria (2006); Martínez (2021)
Phenomenology (Western)	*Epoché*, *Reduction*, *Embodied Reflection*	Suspension of judgment; direct observation of experience beyond mental filters	Husserl (2012); Merleau-Ponty (1962); Giorgi (1983)
Existential/Humanistic Psychology	*Self-transcendence*, *Peak experience*, *Authenticity*	Detachment from ego defenses to encounter meaning and presence	Maslow (1971); May (1981); Rogers (1961)
Contemplative Psychology	*Re-perceiving*, *Mindfulness*, *Meta-awareness*	Observing internal states as objects of attention; emotion regulation	Shapiro et al. (2006); Bishop et al. (2004); Dorjee (2016)
Transpersonal Psychology	*Observing Self*, *Ego-transcendence*	Awareness beyond ego-based identity; access to intuitive and spiritual dimensions	Deikman (1982); Assagioli (1974); Wilber (1997); Wade (1996)
Cultural Psychology/Semiotic Theory	*Symbolic Mediation*, *Internalization*	Detached observation shaped by cultural tools and signs	Vygotsky (1978); Valsiner (2007); Zittoun and Gillespie (2015)
